# TNIP1 in Autoimmune Diseases: Regulation of Toll-like Receptor Signaling

**DOI:** 10.1155/2018/3491269

**Published:** 2018-10-03

**Authors:** Rambon Shamilov, Brian J. Aneskievich

**Affiliations:** ^1^Department of Pharmaceutical Sciences, University of Connecticut, Storrs, CT 06269-3092, USA; ^2^Graduate Program in Pharmacology & Toxicology, University of Connecticut, Storrs, CT 06269-3092, USA; ^3^Stem Cell Institute, University of Connecticut, Storrs, CT 06269-3092, USA

## Abstract

TNIP1 protein is increasingly being recognized as a key repressor of inflammatory signaling and a potential factor in multiple autoimmune diseases. In addition to earlier foundational reports of TNIP1 SNPs in human autoimmune diseases and TNIP1 protein-protein interaction with receptor regulating proteins, more recent studies have identified new potential interaction partners and signaling pathways likely modulated by TNIP1. Subdomains within the TNIP1 protein as well as how they interact with ubiquitin have not only been mapped but inflammatory cell- and tissue-specific consequences subsequent to their defective function are being recognized and related to human disease states such as lupus, scleroderma, and psoriasis. In this review, we emphasize receptor signaling complexes and regulation of cytoplasmic signaling steps downstream of TLR given their association with some of the same autoimmune diseases where TNIP1 has been implicated. TNIP1 dysfunction or deficiency may predispose healthy cells to the inflammatory response to otherwise innocuous TLR ligand exposure. The recognition of the anti-inflammatory roles of TNIP1 and improved integrated understanding of its physical and functional association with other signaling pathway proteins may position TNIP1 as a candidate target for the design and/or testing of next-generation anti-inflammatory therapeutics.

## 1. Introduction

Autoimmune diseases are chronic, relapsing disorders characterized by immune dysregulation featuring loss of tolerance, generation of autoreactive T and B cells, circulating autoantibodies, and chronic inflammation. For those pathologies with genetic variant or expression level differences in the anti-inflammatory protein TNIP1 (TNF*α*-induced protein 3- (TNFAIP3-) interacting protein 1), this can be associated with increased immune cell activation and infiltration leading to tissue-specific defects including but not limited to loss of serum protein to the urine (lupus nephritis), failure of the epidermal barrier (psoriasis), or diminished lung function (systemic sclerosis). These cases occur in the larger cohort of autoimmune diseases. When considered together, these include about 30 diseases with an estimated prevalence of ~9% which in turn is modulated by gender, age, and ethnicity [1, 2]. Compounding these numbers, the incidence of autoimmune disease in westernized countries appears to be increasing [3, 4]. Costing over 100 billion dollars in healthcare expenses and as a leading cause of morbidity especially in women under sixty-five [5], autoimmune diseases are devastating to patients. While major histocompatibility complex (MHC) genes [6] are central to many of these diseases, environmental factors including diet, UV irradiation, drug exposure, and infectious agents [3, 7] as well as numerous non-MHC susceptibility loci [8] are being recognized as players in the complex etiology of autoimmune diseases. Treatment for autoimmune disease has revolved around managing immune-mediated hyperactivity by dampening inflammatory responses and immune cell proliferation. However, this approach leaves patients vulnerable to opportunistic infections which can be life-threatening [9]. Thus, there is a continuous need to discover and define potential inflammatory signal suppressors, some of which are present in those disease-associated non-MHC loci, whose deficiency or dysfunction could contribute to autoimmune disease. There is significant building evidence that TNIP1 (also known as ABIN-1, Naf1, and VAN) [10–12] fits this description. As a suppressor of inflammatory signaling downstream of Toll-like receptors (TLRs), TNIP1 could play a pivotal role in specific autoimmune diseases. TNIP1 genetic association with certain autoimmune diseases and its negative regulation of inflammatory signaling will be explored in this review. Mechanistic understanding of signaling regulators such as TNIP1 could lead to them becoming therapeutic targets.

## 2. TNIP1 (TNFAIP3-Interacting Protein 1)

### 2.1. Genome-Wide Association and Expression-Regulating Studies: Implications in Inflammatory Disease

Over the last decade, TNIP1 has been reported among the highest scoring non-MHC genes across multiple genome-wide association studies (GWAS), spanning multiple populations and diseases including psoriatic arthritis [13–16], systemic sclerosis [17, 18], systemic lupus erythematosus [19–24], and psoriasis [25–27]. TNIP1 sequence variations (single nucleotide polymorphisms (SNPs)) in these populations implicate it in what are nevertheless diseases with likely multiple genetic and environmental factors. However, even with the identification of the heritability of autoimmune disease through twin and familial studies [28, 29], no single gene has been established as the culprit. In common with other disease-associated SNPs [30], those identified for TNIP1 are more likely intergenic and intronic [16, 18, 23, 26]. In general, such nucleotide changes may lessen transcription factor binding at a gene promoter, mRNA processing efficiency, and/or mRNA half-life, leading to decreased protein [31]. Additionally, two microRNAs, miR-517a/c, targeting TNIP1 message, significantly decrease TNIP1 protein levels when transfected in HEK293 cells [32]. In contrast, an apparently phenotypically silent SNP in the TNIP1 mRNA 3'UTR can reduce the negative effect of miR-517a/c. Together, these studies suggest multiple processing and turnover events that contribute to TNIP1 message abundance where those leading to decreased steady-state levels would allow for greater activation of NF-B and hyperresponsiveness to TLR stimuli. While such laboratory investigations functionally link TNIP1 protein levels to the regulation of inflammatory pathway signaling, more study is required to determine what expression steps (mRNA processing, mRNA and protein half-life) are normally or pathologically contributing to these levels.

Parallel to genetic studies, expression microarray experiments have implicated TNIP1 in disease pathogenesis although seemingly paradoxically as its increased transcription was reported for the inflammatory diseases of rheumatoid arthritis and psoriasis [26, 33]. Increased TNIP1 transcription also occurs in B cells following the occupation of cell surface CD40 [34]. These results are actually consistent with positive regulation of TNIP1 expression by NF-*κ*B and its corresponding binding sites in the human TNIP1 gene promoter [35, 36] and the activation of B cell NF-*κ*B post CD40 occupation [37]. However, such mRNA increases are not universally predictive of disease state protein levels as Chen et al. showed decreased TNIP1 protein in psoriatic plaques [38] consistent with loss of its repressive effect and promotion of inflammatory skin disease. In complementary studies, we showed overexpression [39] of TNIP1 protein in HaCaT keratinocytes that led to decreased expression of multiple inflammation-associated genes including interleukin (IL)-6 while TNIP1 reduction [40] promoted expression of numerous cytokine and chemokine genes. Together, these studies demonstrate TNIP1 as an important inflammatory signal response and regulator gene in diverse cell types. It also corroborates the spontaneous systemic autoimmunity observed with TNIP1 deficiency or loss-of-function mutations characterized in part by increased NF-*κ*B activation [41, 42].

### 2.2. Cellular Location and Role in Cell Activation

TNIP1 is found ubiquitously throughout the body in both the nuclear and cytoplasmic compartments of cells [43] where it has been implicated as a mediator of multiple pathways. For example, it functions in the nucleus where it can act as a corepressor of ligand-bound retinoic acid receptors (RARs) [44] and peroxisome proliferator acid receptors (PPARs) [45]. TNIP1 is found in the cytoplasm as well where it is able to interact with HIV-encoded proteins nef [11] and matrix [12], modulate signaling downstream of epidermal growth factor receptor (EGFR) via interactions with ERK2 [46], and interact with the ubiquitin-editing protein TNFAIP3 (alias A20 with roles in inflammation and autoimmunity that have been reviewed elsewhere [47]). The interaction between A20 and TNIP1, mediated by the ABIN-homology domain 1 (AHD1) domain of TNIP1 [48], promotes negative regulation of MAPK activation as well as NF-*κ*B-mediated gene transcription downstream of TNFR and TLRs [10, 42]. TNIP1 function as a negative modulator downstream of select cell membrane receptors and its loss or dysfunction could lead to initiation and perpetuation of an autoimmune phenotype.

### 2.3. Identification of Ubiquitin-Sensing Domain: Importance in Mediating Inflammation

**Figure 1:**
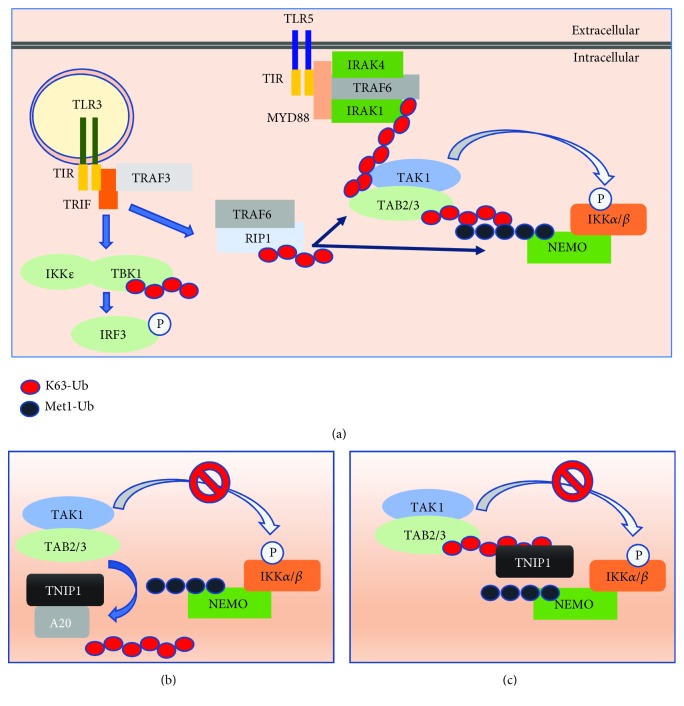
Toll-like receptor signaling—regulation by TNIP1. (a) With TLR dimerization and recruitment of adaptor proteins at the membrane level, ubiquitination of target proteins promotes activation of kinase activity and complex formation. Diverse consequences occur depending on the TLR activated. In the case of TLR3-TRAF3-TRIF, TBK1 becomes ubiquitinated and, in combination with IKK*ε*, promotes phosphorylation of transcription factors (IRF3) upstream of interferon secretion. TLR3 activation may also promote RIP1 ubiquitination, which allows for RIP1 to interact with TAK1/TAB2/3 or NEMO, resulting in gene transcription events regulating inflammation and apoptosis. IRAK1 becomes ubiquitinated followed by TAK1 activation with TAB2/3 binding K-63 linked ubiquitin which forms a hybrid complex with linear (Met1-linked) ubiquitin on NEMO allowing for TAK1 to phosphorylate IKK*β*. This eventually results in the release of NF-*κ*B subunits from I*κ*B*α*. TNIP1 regulation of these events is believed to occur by (b) removal of K63-linked ubiquitin chains via TNIP1/A20 binding and A20 de-ubiquitinase activity and/or (c) inhibition of complex formation by competition for polyubiquitin binding.

TNIP1 does not possess enzymatic activity and is thought to influence intracellular signaling through association with its binding partner ubiquitin-editing enzyme A20 (alias TNFAIP3) [49, 50]. The TNIP1-A20 complex then utilizes the ubiquitin-binding domain of ABIN and NEMO (UBAN, alias AHD2) within TNIP1 for the recognition of linear (Met1) and K62-linked polyubiquitin chains [48]. The homologous UBAN domains also occur in TNIP2, TNIP3, and optineurin with a presumed similar role in the control of cytoplasmic signaling. Expected UBAN functionality stems from earlier reports that polyubiquitin binding by NEMO was a key to NF-*κ*B-mediated transcription downstream of TNF*α* [51–53]. Current understanding of polyubiquitin in signaling downstream of TLRs (among other receptors) describes two roles of polyubiquitin. Firstly, polyubiquitin acts as an activator of kinases by inducing conformational changes in those enzymes when bound (e.g., TAB2/TAB3 binding K63-ubiquitin then activating TAK1 within the TAB2/3/TAK1 complex). Secondly, polyubiquitin may act as a scaffold for colocalization of different complexes associated with TLR activation (e.g., TAB2/TAB3/TAK1 complex activation of the NEMO/IKK*α*/*β* complex through K63/Met1 hybrid polyubiquitin chains) [54] (Figure 1(a)). These interactions promote eventual phosphorylation of targets including MAPKs and the inhibitor of NF-kappa B*α* (I*κ*B*α*) [55]. Initial studies described TNIP1 as capable of increasing the rate of A20-mediated removal of polyubiquitin; with decreased expression of TNIP1 via siRNA knockdown, the rate of A20-mediated de-ubiquitination of NEMO was decreased [56]. However, more recently it has been shown that knock-in mice with A20 mutants featuring no de-ubiquitinase activity presented phenotypically normal and showed normal responsiveness to both TLR (LPS) and TNFR (TNF) ligands [57]. TNIP1 has also been shown to function in signal repression even in the absence of A20 [41, 58]. In the latter study, floxed alleles of A20 and TNIP1 (ABIN-1) in a villin-ER/Cre + mouse system were used to examine intestinal epithelial cell responses to tamoxifen-induced TNIP1 and/or A20 deletion. With the loss of A20 alone, TNIP1 seems to function independently and provide some compensatory response limiting the extensive mortality observed with the dual A20/TNIP1 knockout. As those investigators concluded, there appears to be a “synergistic, though asymmetric, relationship” between A20 and TNIP1 (ABIN-1) [58]. This points to TNIP1 promoting repression of signaling not only due to increased A20 ubiquitin-editing activity but also due to a potential mechanism where TNIP1 disrupts scaffold formation dependent on polyubiquitination and complex formation through binding and prevention of protein-protein interactions (Figures 1(b) and 1(c)**)**.

Aside from NEMO, other targets of polyubiquitin downstream of TLR activation have been described as negatively regulated by TNIP1 including TANK-binding kinase 1 (TBK1) [59], receptor-interacting serine/threonine kinase 1 (RIP1 or RIPK1) [41], and interleukin-1 receptor-associated kinase 1 (IRAK1) [42]. Association of TNIP1 with IRAK1 was abolished with a loss of function mutation in its UBAN domain despite the presence of TLR4 agonist LPS. Loss of UBAN function promoted increased IRF3-mediated transcription downstream of K63 ubiquitin target TBK1 in the presence of TLR3 agonist. RIP1, another TNIP1 interaction partner, participates in signaling downstream of both TLR and TNF receptors, mediating inflammatory responses [60] and cell-death signals [61]. Loss of TNIP1 UBAN functionality (QQ477EE mutation) prevents this interaction and associated regulation of programmed cell death (PCD) [41]. More recently, RIP1 regulation by TNIP1 has been determined to be dependent on both Met1 ubiquitination and K63 ubiquitination, ultimately promoting de-ubiquitination of RIP1 by a more stable A20 [62]. The importance and widespread effects of TNIP1 repression on inflammatory pathways are clearly based on diverse targets of TNIP1-mediated regulation described above. Beyond inflammatory signaling pathways, polyubiquitination affects structure, function, and stability of almost innumerable proteins and thus the related biological consequences of those proteins [63]. We suggest that future studies of TNIP1 interacting with these ubiquitinated proteins are likely to reveal the new and wide-reaching significance of the TNIP1 protein.

## 3. Toll-like Receptors (TLR)

### 3.1. Structure and Role in Cell Activation

Following exposure to microbes, cellular mechanisms are activated to recognize these foreign pathogens and eliminate them. These mechanisms rely on both innate and adaptive immunity through recognition by antigen-specific and nonspecific receptors. During innate immunity, foreign pathogens are recognized by pattern-recognition receptors (PRRs) which include Toll-like receptors, a family of type 1 transmembrane receptors capable of forming hetero- or homodimers on the cell membrane (TLR1, 2, 4, 5, and 6) and intracellularly (TLR3, 7, 8, and 9) within endosomes, lysosomes, or the endoplasmic reticulum [64]. TLRs are commonly expressed on sentinel inflammatory cells such as macrophages and dendritic cells as well as on nonimmune cells such as fibroblasts and epithelial cells [65]. As a component of the innate immune system, TLRs allow for a rapid response to environmental triggers in defense against microbial infections [66]. These receptors are capable of recognizing environmental cues, known as pathogen-associated molecular patterns (PAMPs), associated with foreign pathogens (bacteria, fungi, parasites, and viruses). TLRs are also capable of recognizing host-derived endogenous ligands referred to as damage-associated molecular patterns (DAMPs) which include free fatty acids, oxidized lipids as well as heat-shock proteins (HSP60 and HSP70) and extracellular membrane (ECM) components released during cell injury and apoptosis [64, 67]. PAMPs recognized by TLRs can be separated into two types based on whether they are sensed externally (e.g., bacterial lipids and lipopolysaccharide (LPS) and flagellin) or within intracellular compartments (e.g., nucleic acids including viral dsRNA, ssRNA, and CpG-DNA).

### 3.2. Key Components and Divergent Pathways

Common to TLR is an extracellular domain featuring leucine-rich repeats (LRRs), which allow for diversity in recognizing agonists, and a cytoplasmic Toll/IL-1 receptor (TIR) domain involved in adaptor protein recruitment associated with receptor activation/dimerization [68]. With binding of PAMP or DAMP by the LRR domain, downstream consequences include secretion of chemokines, proinflammatory cytokines, and/or type I/II interferons. This is mediated by a number of recruited TIR domain-containing proteins (Figure 1(a)) including myeloid differentiation primary-response protein 88 (MyD88), TIR-domain-containing adaptor protein inducing *β* interferon (TRIF), TIR-domain-containing adaptor molecule (TIRAP), or TIR-domain-containing adaptor molecule (TRAM). Excluding TLR3, all other TLR initiate signaling by recruiting MyD88 (with TLR1, 2, 4, and 6 recruiting intermediate adaptor protein TIRAP along with MyD88) [69]. TLR3 acts through recruitment of TRIF, with TLR3 requiring secondary adaptor protein TRAM. Although shared signaling events such as phosphorylation and ubiquitination occur, there is a divergence in the use of receptor adaptor protein MyD88.

#### 3.2.1. MyD88-Dependent Signaling Pathway

Loss of MyD88 expression has been associated with decreased ability to mount an immunological response to certain types of infections in mice and humans [70–72]. MyD88 acts as a key bridge between the death domain (DD) containing IL-1R-associated kinase (IRAK) 4 and the TIR domain of TLRs. IRAK-4, a serine/threonine kinase, drives signaling by promoting the activation of two other IRAK proteins, IRAK-1 and IRAK-2, forming what is called the Myddosome [73]. Due to its importance in signaling, efforts have been ongoing to target IRAK-4 therapeutically [74]. With activation of IRAK-1 and IRAK-2, the IRAK proteins dissociate and form a complex TNFR-associated factor 6 (TRAF6) which acts as an E3 ligase in concert with E2 ubiquitin-conjugating enzyme complex UBC13 and UEV1A, promoting auto-K63-linked ubiquitination and activation of mitogen-activated protein kinase kinase kinase 7 (TAK1). TAK1 plays a central role in the activation of both canonical pathways resulting in NF-*κ*B-mediated transcription and noncanonical pathways leading to AP-1-mediated transcription via ERK/JNK/P38 [69]. TAK1 activation is believed to occur following TAK1 complex formation with TAK1-binding protein 1 (TAB1), TAB2, and TAB3. This complex formation (Figure 1(a)) is dependent on TAB2/3 interaction with K63-linked polyubiquitin [75]. Activation of TAK1 promotes further activation of the I*κ*B kinase (IKK*β*) and MAP kinase kinase 6. IKK*β* is associated with IKK*α* and NEMO (known as IKK*γ*) and leads to phosphorylation, K48-linked ubiquitination of NF-*κ*B inhibitor alpha (I*κ*B*α*), and proteasomal degradation freeing NF-*κ*B subunits to translocate into the nucleus. NEMO association with linear polyubiquitin via the linear ubiquitin chain assembly complex (LUBAC) is a key to the eventual I*κ*B*α* degradation via the IKK complex (IKK*α*, IKK*β*, NEMO) [76]. Compartmentalized TLR7 and 9 are distinct from other MyD88-dependent TLRs in that they can promote interferon secretion (mainly IFN*α*), making them powerful tools against invading microbe-associated nucleic acids. This occurs through IRF7 activation by IRAK-1 phosphorylation in a MyD88, IRAK-1, TRAF3, IKK*α*, and IRK7 complex [77].

#### 3.2.2. Non-MyD88-Dependent Signaling Pathway

TLR3 signaling was reported as one of the two TLRs sensitive to TRIF-deficiency in mice [78]. MyD88 deficiency in bone marrow-derived macrophages (BMDM) promoted increased TLR3-mediated cytokine secretion [79]. Through TRIF, TLR3 is capable of inducing TRAF6-mediated gene expression, as in MyD88-dependent signaling, by promoting K63-linked ubiquitination of RIP1 (Figure 1(a)), with pellino1 being a key in the activation of RIP1 [80]. RIP1 can then promote activation of the TAK1 complex and subsequent events leading to I*κ*B*α* degradation. TRAF3, an E3 ligase like TRAF6, promotes TRAF6-independent [81] IRF3 phosphorylation by ubiquitination of TANK-binding kinase 1 (TBK1) and formation of IKK*ε*/TBK1 complex. Activated IRF3 forms a dimer and enters the nucleus where it promotes type I IFNs secretion.

## 4. TNIP1 in Inflammation and Disease

With all the unknown factors remaining in regard to the initiation of autoimmune or hyperinflammatory conditions, it is clear that there are multiple simultaneous factors contributing to these diseases. One such factor is a genetic component such as DNA sequence variants found in association with specific disease states. As presented below, *TNIP1* is an oft-cited gene in GWAS studies with SNPs in certain populations suffering from systemic lupus erythematosus, psoriasis, and systemic sclerosis [13–16]. For recent consideration of possible genetic association of TNIP1 with other autoimmune diseases such as Sjögren syndrome and psoriatic arthritis, the reader is directed to [82, 83].

### 4.1. Systemic Lupus Erythematosus

**Figure 2:**
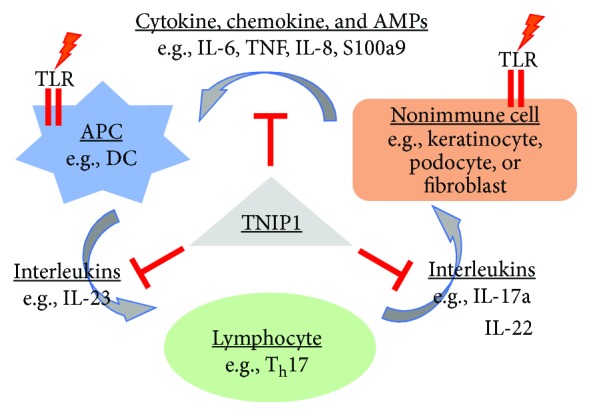
IL-23/Th17 axis of psoriasis. Proposed paracrine signaling with TNIP1 regulation in psoriasis where nonimmune cells are keratinocytes [111]. We would suggest that variations of this could be relevant for SLE and SSc, where the nonimmune cells are a podocyte or fibroblast, respectively. For instance, following TLR7 or 9 activation of APCs in SLE, paracrine signaling would include type I interferons.

In SLE, the loss of immune tolerance and with it, triggering of autoreactive T and B cells appears as a key to the development and progression of the disease state which is compounded by genetic predispositions and exposure to environmental risk factors. Plasmacytoid dendritic cells (pDCs) are antigen presenting cells (APC) capable of sensing ssRNA and unmethylated CpG DNA sequences through endosomal TLR7 and 9, respectively [84]. When activated by these ligands, pDCs produce high levels of type I interferons (IFN*α*/*β*) (Figure 2). IFN*α* has been implicated in many clinical manifestations of SLE [85] and, interestingly, when used therapeutically, IFN*α* has been shown to induce a SLE-like phenotype [86]. pDC activation by host DAMPs is avoided as these cells can distinguish between microbial and self-nucleic acids [87]. However, such tolerance is believed to be compromised with generation and accumulation of increased protein and nucleic acid associated DAMPs from apoptosis-associated proteases and nucleases. Circulating pDCs internalize these new DAMPS with complexes formed with IgG leading to potent activation of endosomal TLR7 and 9 [88, 89] and increased IFN*α* production [90–92]. TLR7 null mice were partially protected from similar effects due to decreased pDC responsiveness and in-turn reduced IFN*α* and IL-6 expression [93]. Conversely, increased expression of TLR7 in transgenic mice overexpressing TLR7 spontaneously developed fatal and acute immune dysregulation and autoimmunity [94]. With these observations in human SLE patients and mice experiments, the importance of TLR activation in the progression of the SLE phenotype becomes clear.

The GWAS correlation of TNIP1 sequence variants to several autoimmune diseases [17–19, 22, 26], for example, and evidence of NF-*κ*B signaling possibly under TNIP1 control post-TLR signaling [10, 19, 26, 50, 95] provided rationale for investigating SLE-like characteristics in knockout or mutant knock-in mouse models, respectively, with missing or dysfunctional TNIP1 protein. Such mouse experimental systems set the stage to study TNIP1 functional differences underlying likely contribution to phenotypes of the whole animal activated immune systems.

To address the high proportion of late embryonic *Tnip1* −/− lethality seen earlier [41], Zhou and colleagues crossed 129S2 ES-cell-derived *Tnip1* +/− mice to a C57BL/6 background [96]. Although the influence of mouse genetic strain remains unresolved, there is an increase in phenotypically normal live-born pups; however, by four months of age, these mice develop a wasting syndrome along with leukocyte infiltration of kidneys, liver, and lungs. Echoing a SLE phenotype, there are increased levels of autoreactive antibodies against dsDNA and development of significant glomerulonephritis which are not rescued by a TNFR null background. In contrast to the expected NF-*κ*B-mediated transcriptional increases, CpG occupation of TLR9 in *Tnip1* −/− bone marrow-derived macrophages [96], strikingly activates C/EBP*β*-regulated promoters including that of proinflammatory SLE severity-correlating S100A8 [97]. Strikingly, knock-in mice engineered with a Tnip1 mutant [D485N] [42] defective in binding both K63-linked and linear polyubiquitin chains (see UBAN section above), and therefore with predicted increased TNF*α* sensitivity, are born at the same size and frequency of *Tnip1* +/+ mice. These *Tnip1* [D485N] mice show age-associated autoimmunity marked by enlarged spleens and circulating levels of antinuclear and anti-DNA antibodies, the latter consistent with the development of severe glomerulonephritis [42]. For cultured cells, Tnip1 [D485N] MEF do not display TNF*α*-enhanced activation of MAPKs but do show exaggerated responses via activation of JNK, p38, and NF-*κ*B in B cells and BMDC exposed to several TLR ligands including LPS (TLR4), lipoteichoic acid (TLR2/6), and R848 (TLR7). Along these lines, when challenged with TNF*α*, human immortalized kidney podocyte cells recombinantly expressing the human ubiquitin-binding deficient homologue mutant [D472N] but not human wildtype TNIP1 [98] have increased expression of numerous chemokine genes echoing increased NF-*κ*B-regulated inflammatory signaling in SLE and glomerulonephritis patient kidney samples. Further complementary studies of both receptors' cytoplasmic activation steps should help determine the full range of TNIP1 protein suppressive capabilities. For instance, in *Tnip1* [D485N] mice, a lupus nephritis-like presentation is prevented when crossed to backgrounds with catalytically inactive signaling proteins downstream of TLRs such as IRAK-1 or IRAK-4 [99].

The range and sometimes divergence of TNIP1 effects on postreceptor signaling interpreted from overexpression studies, whole animal null or knock-in, and cell-specific deletion [100–103] may be reflecting its as yet uncharacterized quantitative and/or qualitative characteristics. These may arise from mouse strain influences, varying expression levels of TNIP1 protein and/or its varying interacting partners in different cells [11, 43, 104, 105] and cell- or organ-specific sensitivity to various cytokine and PAMP/DAMP receptor agonists. Further study in these complementary systems will help to advance our understanding of a role for TNIP1 in SLE pathology. Reconciling such differences will be central to considering any possible therapeutic targeting of TNIP1's repression of the multiple signaling pathways downstream of distinct membrane receptors.

### 4.2. Psoriasis

Psoriasis is a chronic skin disorder affecting about 2% of people in the United States, most commonly manifested as plaque psoriasis (psoriasis vulgaris) and accounting for about 90% of all cases. Psoriasis is an immune-mediated disorder that manifests in the skin or joints, which is influenced by both environmental and genetic components [106]and characterized by aberrations in the skin epithelium specifically hyperproliferation in the epidermis and hyperactive keratinocytes with increased mitotic rates. This increased replication of keratinocytes results in poor maturation leading to incomplete cornification observed clinically as poor barrier function and histologically as retained nuclei in the stratum corneum (parakeratosis) and thickening of the epidermis (acanthosis) with elongated rete ridges. Immune infiltration is a hallmark of psoriasis featuring increased dendritic cells (DCs) and macrophages in the dermis and neutrophils in the epidermis. T cells are found in increased numbers in both with higher numbers of Th1, Th17, and Th22-polarized T cells [107]. Family-based linkage studies have established the strongest genetic links to psoriasis with the class 1 region of the MHC cluster near the HLA-Cw6 allele. These studies are consistent with GWAS studies which have identified the same HLA region as well as other genes including IL-23R, IL-12*β* (p40), and TNIP1 [26, 108, 109].

Differential TNIP1 expression in psoriatic patients versus healthy controls has been described previously [26] with observed increases in mRNA expression coupled with a 2-fold decrease in protein expression in the patients [38]. To establish models of TNIP1 loss in psoriasis, various cell culture models and in vivo approaches have been taken. Chen et al. used TNIP1 shRNA injection to promote local TNIP1 deficiency in a region of the skin followed by topical exposure to the TLR7 agonist imiquimod (IMQ), which is a compound used frequently to promote psoriasis-like phenotype in mice [110]. In these mice, tissue with reduced TNIP1 had a significantly higher overall psoriasis disease score, as compared to wild-type IMQ treated. This included increased raised, scaly, erythematous plaques marked by epidermal thickening, hyperkeratosis, and parakeratosis. These characteristics closely mimic the presentation of psoriasis in human patients. Using a TNIP1 null line challenged with IMQ, Ippagunta et al. demonstrated that skin inflammation has more characteristic of psoriasis than atopic eczema via comparison with microarray analysis of corresponding human disease samples [111]. These mouse models suggest that human disease may be a combination of genetic predisposition (TNIP1 SNPs) coupled with environmental assault (TLR ligands).

Psoriasis is believed to be a disease of both epithelial cell dysfunction coupled with immune cell over-activation. This mechanism centers around T Helper (Th) 17 cells which secrete IL-17*α* and IL-22, potent inducers of keratinocyte activation and proliferation [112]. Interestingly, cultured cells experimentally exposed to IL-17a showed increased rates of TNIP1 protein degradation [113]. IL-23 secreted by activated DCs promotes the expansion of already differentiated Th17 cells (from naive CD4+ T cells in the presence of TGF*β*, IL-1*β*, and IL-6) [114, 115]. Increased keratinocyte activation promotes increased chemokine, cytokine, and antimicrobial peptide secretion resulting in increased DC activation leading to increased IL-23 which promotes Th17 proliferation leading to subsequent keratinocyte activation and completing the cycle (Figure 2) [112, 116]. Most of the mechanistic understanding of psoriasis is in the propagation and worsening of the disease state. Still lacking is an understanding of what promotes initiation of the disease. One proposed mechanism for initiation begins with external stimuli such as microbial infections or physical trauma leading to RNA/DNA-LL-37 (antimicrobial peptide) complex formation and internalization by circulating pDCs, where it acts as an agonist for endosomal TLRs 7 and 9, resulting in IFN*α* secretion. IFN*α* expression promotes DC activation which would allow for antigen presentation, T cell differentiation, etc. [106]. Under normal conditions, this would lead to an appropriate immune response and activation. Under conditions with genetic polymorphisms in *PSORS1*, *IL-23R*, *A20*, *TNIP1*, and/or other genes implicated in association studies, this response may become exaggerated. Our group and others have shown that TNIP1 reduction alone does not promote a chronic hyperinflammatory state [38, 40]. However, when coupled with an external stimulus, there is a clear exaggerated response [38, 40]. Thus, keratinocytes deficient for TNIP1 may contribute to hyperactivation of local immune responses when exposed to TLR environment- or skin cell-derived agonists.

For studies of cell signaling in a psoriasis-relevant model, Callahan et al. used CD11c-Cre LoxP system to engineer a TNIP1 DC-specific knockout [103]. When exposed to TLR ligands, these mice developed splenomegaly and lymphadenopathy with an accumulation of myeloid cells suggesting a systemic activation of the immune system, as previously observed in the whole mouse TNIP1 knockout [41, 96]. Topical exposure to IMQ, a TLR7 ligand, instigated a higher overall psoriasis phenotype score (higher levels of epidermal hyperplasia, hypogranulosis, hyperparakeratosis, and epidermal neutrophil infiltration) compared to control animals. BMDC from these mice when treated with TLR ligands (IMQ, LPS) showed increased secretion of immune cytokines including IL-23, an interleukin considered critical in psoriasis development due to its ability to induce Th17 cell proliferation. Ippagunta et al., confirmed this finding using BMDC from TNIP1 knockout mice. They also demonstrated the importance of TNIP-mediated regulation of TLRs in T cell and nonimmune cell (keratinocytes, fibroblasts) mediated immunity [111]. Using keratinocyte-specific TNIP1 knockout mice, these investigators demonstrated that keratinocyte-initiated immune signaling, through the IMQ-induced expression of numerous cytokines, antimicrobial peptides, and chemokines, can induce psoriasis-like disease. Thus, for several immune and nonimmune cell types, these studies demonstrate the importance of TNIP1 control over internal signaling pathways and also external paracrine interactions among them (Figure 2).

### 4.3. Systemic Sclerosis (Scleroderma)

The etiology, clinical pathology, and cell biology of systemic sclerosis (SSc) lead to multicell signaling interactions coming together to trigger micro-vascular damage, inflammatory signal production, and cardiac, pulmonary, and dermal fibrotic responses [117]. Prevalence of SSc cases (~14 per 100,000 persons) can vary significantly as there appear to be significant disease subtype, gender, age, race, and ethnic group qualifiers [118, 119]. While thickening, hardening, and tightening of the skin are classic and common in most presentations, it is a decline in internal organ function, such as the lungs and gastrointestinal tract from local vasculopathy and excess extracellular matrix deposition that most significantly promotes disease morbidity and mortality [120–122].

Familial and epidemiologic studies provide clear genetic associations between SSc and HLA [117, 123–125] and non-HLA loci which are often in-common for other autoimmune pathologies [126]. Three separate reports have added TNIP1 to these non-HLA loci [17, 18, 127]. Like many pathology-associated SNPs, TNIP1 sequence variations associated with SSc to date are in noncoding sequences (intronic and potential genetic expression-regulatory regions). Interestingly, three other proteins with proven physical interaction with and/or signaling regulatory control by TNIP1 are implicated in SSc by GWAS reports suggesting their mutual contribution to relevant pathways. A PPAR*γ* SNP [128, 129] is associated with SSc, and we previously established TNIP1 as a nuclear corepressor of PPAR*α* and *γ* activity [45]. At least three SNPs of the TNIP1 cytoplasmic binding partner TNFAIP3 (A20) carry an increase for susceptibility to SSc [117, 130–132]. Additionally, the SNP resulting in an amino acid substitution (Pro631His) in TLR2 is associated with increased progression of SSc-associated pulmonary arterial hypertension [133, 134]. Thus, even when not a genetic variant itself, signal control/initiation node (s) (TNFAIP3, TLR, and PPAR) impacted by TNIP1 may be contributory to SSc.

In addition to genetic sequence variants for the TNIP1 pathway-associated proteins TLR and TNFAIP3, several functional and expression levels studies have linked their normal counterparts to SSc. In part because of the relative clinical accessibility of cutaneous biopsies compared to internal organ sampling, these studies may be biased to dermal fibroblast characteristics. Nevertheless, several studies have found expression of TLR9 [135] and TLR4 as well as endogenous ligands for the latter [136–138] increased in SSc lesion specimens. Fibroblasts cultured from SSc skin biopsy explants displayed a degree of TLR3 expression induction by interferon greater than control cells. Additionally, SSc lesion-derived dermal fibroblasts, under otherwise standard culture, demonstrated a sensitivity to the TLR3 synthetic ligand poly (I:C) greater than control fibroblasts [139]. The full spectrum of TLR in multiple cell types and their demonstrated or possible involvement in SSc has been comprehensively reviewed by Fullard, Bhattacharyya, and colleagues [136, 140]. Thus, deficiency or dysfunction in TNIP1-adjacent proteins such as its functional partner TNFAIP3 (A20) or excess upstream signaling initiated by sensors of innate immune system activity such as TLR may overwhelm the signaling-dampening abilities of TNIP1 [134, 141].

Intriguingly, following up on TNIP1 SSc SNPs [18], Allanore and colleagues reported that compared to skin samples derived from age- and sex-matched healthy controls, the TNIP1 protein was decreased in SSc lesional skin. Separate expression array studies [142] support reduced TNIP1 in lesional skin compared to patient uninvolved skin. Cultured SSc patient dermal fibroblasts [18] have reduced TNIP1 mRNA and protein. These SSc fibroblasts produced elevated levels of collagen in response to cytokine challenge (TNF). Strikingly, this could be abrogated by extracellular TNIP1 protein. This effect would have had to be through an unknown mechanism versus characterized intracellular signaling repression by TNIP1. There is no known or predicted cell-penetrating peptide-like region for cellular reentry of TNIP1. Other reports and our publications established cytoplasmic and nuclear compartmentalization for TNIP1 from endogenous and transfected cDNA expression studies [12, 43]. Nevertheless, the studies establishing the TNIP1-SSc genetic association and/or the reduced TNIP1 expression levels in SSc samples [17, 18, 127, 142] are consistent with and supportive of TNIP1 as a key suppressor of pathways driving a fibrotic signaling whether intrinsic to the fibroblast or in a paracrine fashion where TNIP1 deficiency or defective function in associated epithelial cell (e.g., skin keratinocytes) could make these otherwise protective cells a source of fibroblast-activating cytokines.

## 5. TNIP1 in Autoimmunity: Summary

Based on the studies of TNIP1 deficiency across a broad spectrum of cell types, including immune cells (DCs, B cells, and T cells) and nonimmune cells (keratinocytes and fibroblasts), it is clear that the loss of TNIP1 expression through knockout or knock-in functional mutations leads to a predisposition for development of autoimmunity. GWAS studies predict this predisposition in human populations through multiple recognized SNPs in TNIP1 genes in patients with autoimmune diseases. Not only does TNIP1 loss or deficiency promotes a hyperinflammatory state, this phenotype closely mimics autoimmune diseases present in human populations. Because of this, manipulation of TNIP1 expression or function can be a useful tool in modeling autoimmunity in mice for the development of drugs and therapies. One fundamental question remaining in autoimmune disease pathogenesis is the cause and initiation of the disease. Through the TNIP1 autoimmune models, it is clear that the potential for TLR stimulation via microbial invasion, cell damage due to wounding, etc. is the first step in pathogenesis. More specifically, this step is described by an exaggerated stimulation of TLRs due to the loss of negative regulation by TNIP1 downstream of receptor activation. With its key regulatory control over multiple signal initiators or pathways, further research on TNIP1 could advance it from association with several autoimmune diseases to a mechanistic contributor to the pathology, and possibly, ultimately, a therapeutic target.

## 6. Conclusions and Future Directions

- The vast majority of autoimmune diseases does not stem from single gene defects and are likely influenced by multiple genes [143] and environmental factors [144] that can promote body-wide and/or tissue-specific autoinflammatory reactions. From human genetic studies, animal models, and in vitro experiments, it would appear that loss, reduction, or dysfunction TNIP1 has the potential to be one of these defects.
- With significant negative side-effects of current autoimmune disease therapies, there is a pressing need to develop safer treatments through research into mechanisms underlying these disorders. As highlighted in this review, TNIP1 protein function, as a repressor of signaling downstream from TLR implicated in several autoimmune diseases, could be a pharmacologic target of new therapies.
- While likely relevant to hyperactive inflammatory signaling in several autoimmune diseases, the experimental loss or reduction of TNIP1 presents as its own phenotype and does not fully recapitulate any one specific pathology. Nevertheless, the predisposition towards hyperinflammatory reactions of multiple cell types with defective TNIP1 function is likely to provide advantageous insights to further study of tissue-specific and whole animal autoimmune disease models as well as testing of new anti-inflammatory therapies.
